# Dysregulated TFEB–autophagy-lysosome pathway links acute COVID-19 immunopathology to Long COVID sequelae

**DOI:** 10.3389/fimmu.2025.1708364

**Published:** 2025-11-28

**Authors:** Magdalena Gabig-Cimińska

**Affiliations:** Department of Medical Biology and Genetics, Faculty of Biology, University of Gdańsk, Gdańsk, Poland

**Keywords:** SARS-CoV-2, COVID-19, Long COVID, autophagy-lysosome pathway (ALP), lysosomal organelle, transcription factor EB (TFEB), host-directed therapy

## Abstract

SARS-CoV-2 disrupts cellular homeostasis, including the autophagy-lysosome pathway (ALP), a critical component of innate immunity and viral clearance. By subverting autophagy, SARS-CoV-2 proteins such as ORF3a, ORF7a, and NSP6 inhibit autophagosome-lysosome (APG-L) fusion, generating “incomplete autophagy” that permits viral persistence and drives hyperinflammation. Transcription factor EB (TFEB), a master regulator of lysosomal biogenesis and autophagy, has emerged as a central player in the host response to coronavirus infection. TFEB orchestrates the expression of genes required for lysosomal function and autophagic flux while also shaping immune processes, including cytokine production, interferon-stimulated gene expression, and inflammasome clearance. This mini review synthesizes current knowledge on the TFEB-ALP axis in COVID-19 pathogenesis, highlighting its influence on acute immunopathology and its potential contribution to post-acute sequelae (Long COVID). Restoring TFEB activity and autophagic flux may counteract SARS-CoV-2 evasion strategies and restrain aberrant inflammatory responses. Harnessing the TFEB-autophagy pathway as a host-directed therapeutic strategy could help rebalance immune homeostasis, limit tissue damage during acute infection, and mitigate persistent inflammatory sequelae in Long COVID.

## Introduction

The coronavirus disease 2019 (COrona VIrus Disease 2019; COVID-19), caused by Severe Acute Respiratory Syndrome-CoronaVirus-2 (SARS-CoV-2), represents the third major human outbreak of coronaviruses, following Severe Acute Respiratory Syndrome, caused by SARS-CoV (SARS, caused by SARS-CoV) in 2002–2003 and Middle East Respiratory Syndrome (MERS, caused by MERS-CoV) in 2013 (1–3). Unlike the earlier epidemics, COVID-19 rapidly escalated into a global pandemic, challenging healthcare systems worldwide and leaving long-lasting consequences for public health. Despite the deployment of vaccines and several antiviral or immunomodulatory drugs, the disease continues to pose threats due to viral evolution, heterogeneous host responses, and the emergence of persistent post-acute sequelae known as Long COVID.

The autophagy-lysosome pathway (ALP) has emerged as a critical host process targeted by coronaviruses. ALP orchestrates cellular homeostasis by regulating degradation, recycling, and innate immune defense. Recent studies consolidate how SARS-CoV-2 rewires the ALP to favor replication and persistence (4). SARS-CoV-2 proteins actively interfere with autophagic flux, blocking autophagosome-lysosome (APG-L) fusion and remodeling endomembranes to favor viral replication. Such viral hijacking leads to “incomplete autophagy,” which not only sustains intracellular persistence of viral components but also amplifies pro-inflammatory cascades.

**Figure 1:**
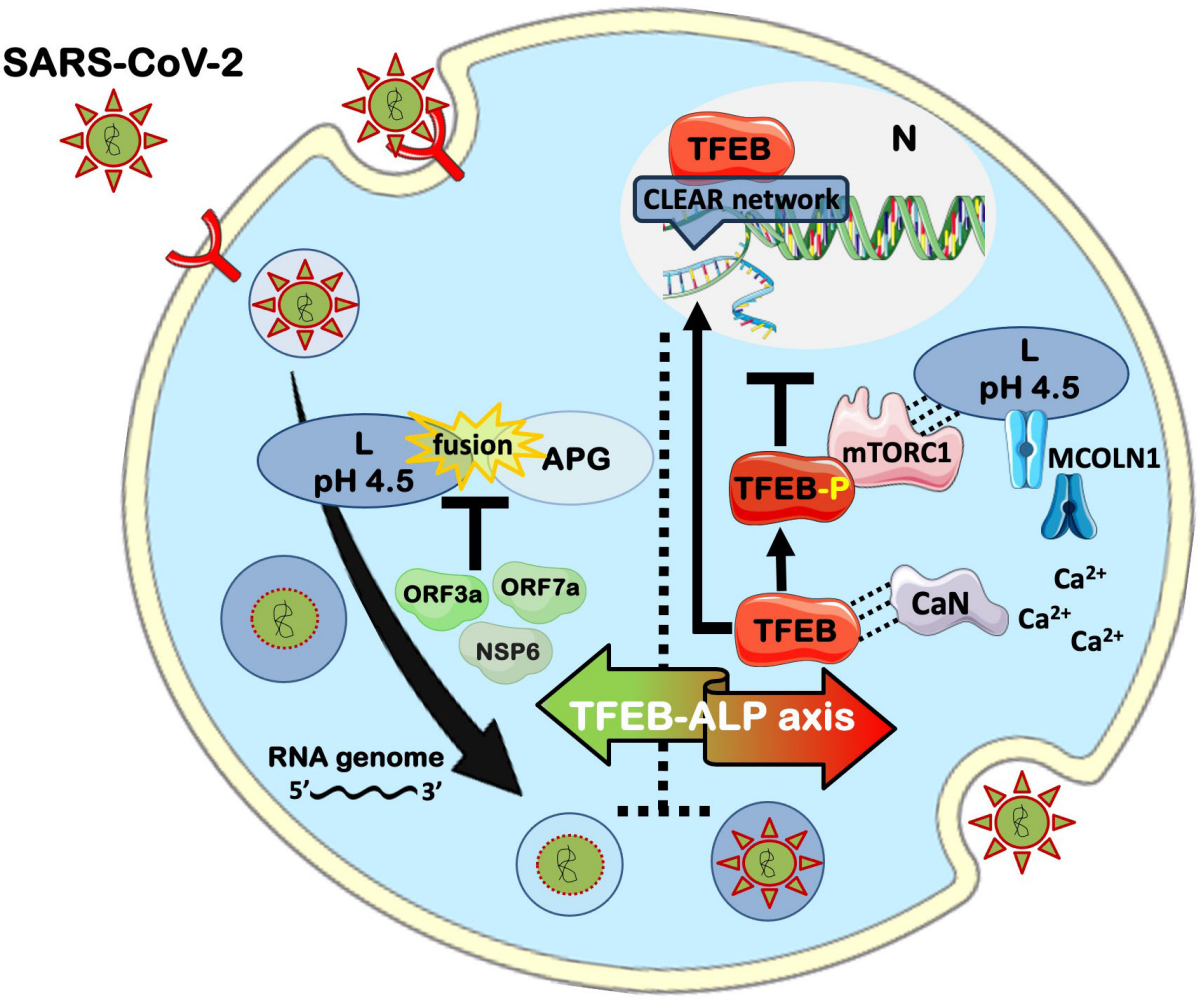
Transcription factor EB-centered control of the autophagy-lysosome pathway (TFEB-ALP axis) during SARS-CoV-2 replication and viral hijacking. Efficient infection proceeds via endocytic entry and low-pH–dependent uncoating in Ls, after which viral RNA replicates and progeny virions can exit by lysosome-dependent exocytosis. At the core of this interface is TFEB, the master transcriptional regulator that determines whether Ls function as degradative organelles or are diverted toward secretion. Because Ls represent the terminal compartment of autophagy, TFEB control extends beyond degradative fate to the entire autophagy-lysosome pathway (ALP). This regulatory hub, referred to as the TFEB-ALP axis, integrates nutrient and stress signals to coordinate autophagic flux and lysosomal biogenesis, thereby shaping both cellular clearance and immune responses. Nutrient sufficiency recruits mTORC1 to the lysosomal surface to phosphorylate TFEB and retain it in the cytoplasm, limiting CLEAR-program transcription. Conversely, under nutrient deprivation or stress, opening of the lysosomal Ca²^+^ mucolipin 1 (MCOLN1) channel triggers calcineurin (CaN) activation, dephosphorylating TFEB and driving its nuclear translocation. Nuclear TFEB restores lysosomal competence – boosting L biogenesis, re-acidification, hydrolase maturation, and terminal autophagic flux (APG-L fusion) – thereby favoring degradation of viral components. SARS-CoV-2 proteins (e.g., ORF3a/ORF7a/NSP6) antagonize this TFEB-directed axis by stalling fusion and dampening cathepsin activity, tilting trafficking toward exocytosis. The schematic highlights this tug-of-war centered on TFEB, whose activation must reestablish degradative dominance without inadvertently enhancing lysosomal egress. *Figure created partially using SERVIER MEDICAL ART (**https://smart.servier.com/**), licensed under a Creative Commons Attribution 3.0 Unported License, and BioRender (*https://app.biorender.com *).* ALP, autophagy-lysosome pathway; APG, autophagosome; APG-L, autophagosome-lysosome; Ca^2+^, calcium ion; CaN, calcineurin; CLEAR, Coordinated Lysosomal Expression and Regulation; L, lysosome; MCOLN1, mucolipin-1 channel; mTORC1, mechanistic target of rapamycin complex 1; NSP6, non-structural protein 6; N, nucleus; ORF3a, open reading frame 3a; ORF7a, open reading frame 7a; SARS-CoV-2, Severe Acute Respiratory Syndrome-CoronaVirus-2; TFEB, transcription factor EB.

Central to ALP control is transcription factor EB (TFEB), a master regulator of lysosomal biogenesis and autophagy genes (5–7). TFEB integrates nutrient and stress signals to maintain homeostasis and immune responses. Recent evidence indicates that TFEB contributes to interferon-stimulated gene expression, cytokine secretion, inflammasome clearance, and antigen presentation (8–12). These functions position TFEB as a pivotal node at the intersection of viral replication and host defense. A schematic overview of these processes, SARS-CoV-2 endolysosomal entry, TFEB gating at the lysosomal surface, and their consequences for autophagic flux is shown in **Figure 1**.

This mini review synthesizes current insights into the viral manipulation of ALP and the immunological role of TFEB in COVID-19. Special emphasis is placed on how disruption of this axis shapes acute immunopathology and may contribute to the persistence of inflammatory signals in Long COVID, highlighting the therapeutic potential of host-directed interventions.

## Viral modulation of the autophagy-lysosome pathway

Coronaviruses have evolved sophisticated mechanisms to exploit the autophagy-lysosome system. In SARS-CoV-2, several accessory proteins subvert ALP at distinct steps, impairing autophagic degradation while preserving structures that support viral replication (**Table 1**).

**Table 1:** SARS−CoV−2 proteins modulating the autophagy-lysosome pathway and their effects.

Viral protein	Primary ALP/lysosome effect (mechanism)	Representative readouts	Implications for pathogenesis/therapy	Key refs
Spike (S)	Entry via endo/lysosomal route under low pH; cathepsin B/L cleavage of S enabling fusion (not an ALP antagonist per se, but places early steps under lysosomal control)	Endosomal entry assays; cathepsin inhibition; low−pH dependence	Rationale for targeting lysosomal pH/trafficking at entry stage; interpret lysosomotropic probe effects cautiously	(22)
ORF3a	Blocks APG-L fusion via interference with HOPS/VPS39; lysosomal dysfunction → “incomplete autophagy”	Accumulation of LC3−II+/p62+ autophagosomes; reduced LAMP1 colocalization; lysosomal alkalinization; inflammasome activation	Maintains viral components outside autolysosomal degradation; exacerbates inflammatory signaling	(13)
NSP6	Restricts autophagosome expansion and remodels ER−derived membranes; modulates autophagy and supports replication organelles	Smaller autophagosomes; altered autophagy flux markers; dependence of replication on NSP6−mediated remodeling	Facilitates replication while blunting degradative autophagy; host−directed strategies could restore flux	(15)
ORF7a	Initiates autophagy yet blocks APG-L fusion via SNAP29 degradation (caspase−3 dependent)	↑LC3 puncta with impaired flux; ↓SNAP29; reduced fusion readouts	Promotes persistence by preventing autolysosomal clearance	(14)
ORF8b (SARS−CoV; homologous context)	Triggers lysosomal stress and TFEB nuclear translocation (evidence from SARS−CoV; informs TFEB axis relevance)	TFEB nuclear localization; lysosomal stress markers	Illustrates that CoV proteins can engage TFEB/ALP signaling directly	(23)

### ORF3a

Open reading frame 3a localizes to lysosomes and interferes with the homotypic fusion and protein sorting complex (HOPS) complex by binding vacuolar protein sorting 39 homolog (VPS39), preventing its association with Ras-related protein Rab-7 (RAB7). The result is a blockade of APG-L fusion and accumulation of unfused autophagosomes. ORF3a-mediated lysosomal dysfunction promotes incomplete autophagy, persistence of viral material, and enhanced inflammasome activity, amplifying inflammatory signaling (13).

### ORF7a

By promoting caspase-3-dependent degradation of SNAP29, Open reading frame 7a limits APG-L fusion. Although autophagy is initiated, the blockade at the fusion step results in impaired clearance of viral components and sustains infection (14).

### NSP6

Non-structural protein 6 restricts the expansion of autophagosomes and remodels endoplasmic reticulum membranes, thereby generating replication organelles. Recent findings indicate interactions between NSP6 and host sigma-1 receptor, implicating this protein in controlling both autophagy and viral replication. NSP6 activity thus supports viral replication while constraining degradative autophagy. Mechanistic updates and phenotypes were summarized in 2023 and extended with cell-biological detail in late-2023 (15).

### Other coronavirus proteins

Evidence from SARS-CoV ORF8b demonstrates induction of lysosomal stress and nuclear translocation of TFEB, linking viral proteins directly to TFEB activation (16). Similar effects have been described for SARS-CoV ORF3a, which promotes lysosomal membrane permeabilization and exclusive nuclear localization of TFEB. These observations suggest that coronaviruses not only inhibit autophagic degradation but may also manipulate TFEB-dependent transcription to alter host defense pathways. In the context of SARS-CoV-2, ORF8 shares partial homology with SARS-CoV ORF8b, and several studies have shown that SARS-CoV-2 ORF8 exploits the autophagy–lysosome system for immune evasion, most notably by promoting autophagy-dependent degradation of MHC-I molecules. However, direct evidence that ORF8 itself triggers lysosomal stress or TFEB activation to the same extent as ORF8b is still lacking; any mechanistic parallels should therefore be regarded as a plausible but unconfirmed hypothesis. More recent work further indicates that ORF8 modulates ER-phagy and endoplasmic-reticulum stress, thereby indirectly shaping autophagy-related pathways (17–19).

Host factors also gate this axis. RAB5 was only recently identified as a host dependency factor for building replication organelles, with NSP6 involved; conversely, Syntaxin-6 (STX6) emerged as a restriction factor that diverts incoming virus toward autophagy-lysosomal degradation – both studies highlight actionable host nodes of ALP trafficking (20, 21). These findings reinforce the centrality of lysosome signaling/biogenesis (TFEB-ALP axis) in CoV-host conflict and motivate host-directed strategies that restore autolysosomal fusion rather than broadly suppressing ALP (13).

The cumulative impact of these viral strategies is the creation of a cellular environment favoring replication and persistence while preventing efficient autophagic clearance. Importantly, such interference also alters immune regulation, as defective autophagy can dysregulate cytokine production and antigen presentation.

## TFEB as a central regulator of ALP and immune responses

Multiple cellular processes are modulated by signaling pathways originating from the lysosomal surface, changing the “old fashioned” view of lysosomes as the degradative endpoint of the endosomal route (24). Lysosome properties, and the role of this organellum as a pivotal signaling hub for cellular metabolism, depend greatly on the mechanistic Target of Rapamycin Complex 1 (mTORC1) activity which is fulfilled through the coordinated interaction of many proteins with mTORC1 at the lysosome surface. When mTORC1 activity is turned on due to the availability of various nutrients, it promotes translation and lipogenesis, while suppressing autophagy. At that point, TFEB, the master regulator of lysosomal biogenesis and autophagy is phosphorylated by mTORC1 at the lysosomal surface and retains in the cytoplasm. When nutrients are scarce or cells are exposed to stress, mTORC1 activity decreases and calcium (Ca) release through the mucolipin 1 (MCOLN1) channel activates phosphatase calcineurin (CaN) which results in TFEB dephosphorylation and, in turn, its nuclear translocation (25). Once in the nucleus, TFEB drives the expression of genes that enhance autophagic flux, lysosomal biogenesis, and metabolic adaptation. On the other hand, when a shortage of metabolic signals occurs the anabolic activities are halted, but autophagy is launched (24, 26). These two mechanisms of induction of autophagy and lysosomal biogenesis, involving mTORC1 and CaN, strongly support the fundamental role of lysosomes in the adaptive response and in the regulation of cellular homeostasis (6, 27, 28). This cautious control of cellular homeostasis, with an important role in cellular defense as, in compromised situations, contributes to the proteolytic degradation of components of invading pathogens and other types of biological cell aggressors (29, 30).

Beyond its canonical role in cellular clearance, TFEB and the lysosomal system also participate in immune regulation (8, 9, 11, 12, 31, 32). Nowadays, the intensively developing field of biology called lysosomics provides a range of important information about the role of TFEB-lysosomes in the inflammatory response and autoimmune conditions (28, 33, 34), among others, though the first signs of lysosomal involvement in inflammatory response were identified decades ago (35). These observations have become increasingly widespread, as multiple studies report on how the disruption of normal lysosomal function leads to abnormalities in inflammation and immunity (31, 32, 34, 36). The adaptative and innate arms of the immune system, both using autophagy and the endolysosomal system in a variety of capacities to mediate immunity, work together to protect host organisms from foreign pathogen invasion. Essential components of the immune response are cellular processes attributed to TFEB activation/overexpression status, similar to its pivotal role in cellular clearance and autophagic flux (8, 29, 37). TFEB, along with microphthalmia-associated transcription factor (MITF), transcription factor E3 (TFE3) and transcription factor EC (TFEC) constitutes a microphthalmia subfamily of basic helix-loop-helix-leucine zipper (bHLH-zip) elements, belonging to the MiT family transcription factors responsible for the regulation of the endolysosomal pathway and the maintenance of autophagic flux controlling cellular metabolism (7). The identification of TFEB as a global modulator of intracellular clearance and energy metabolism, through the regulation of genes involved in the ALPs, has provided new insights into the mechanism by which the cell responds to environmental cues such as malnutrition (5). It has been shown to induce interferon-stimulated genes and to promote the expression of cytokines and chemokines necessary for antimicrobial defense (10). TFEB activity contributes to the autophagy-dependent secretion of cytokines and to the clearance of inflammasomes, thereby modulating innate immune tone. In macrophages, TFEB activation is essential for the rapid transcription of proinflammatory mediators following pathogen exposure (8). Similar links between autophagy-related pathways, cytokine regulation and chronic inflammation have been reported in psoriasis (38). Evolutionarily, its role in host defense is conserved, as demonstrated by HLH-30, the TFEB ortholog in *Caenorhabditis elegans*, which orchestrates antimicrobial gene expression during infection. HLH-30 becomes activated during infection and shuttles from the cytosol to the nucleus of most cells in the *C. elegans* organism (8).

TFEB also impacts adaptive immunity through its influence on antigen presentation (8, 9, 11). By controlling lysosomal activity, TFEB regulates the processing of exogenous antigens and their presentation on MHC class II molecules. Proper regulation of this pathway ensures efficient activation of T cells while preventing aberrant immune activation. Dysregulated TFEB activity, in contrast, can impair antigen presentation and contribute to immune imbalance, indirectly promoting immune activation. In this way, the pH increase inside the lysosome negatively influences antigen-presenting cells (APCs), such as plasmacytoid dendritic cells (pDCs) and B cells (39).

As a master regulator of autophagy, TFEB may also modulate virus replication, promoting virophagy (the degradation of cytoplasmic viral components). On the other hand, the positive-strand RNA viruses such as SARS-CoV-2 use autophagy to facilitate viral replication, since the maturation of autophagosomes is needed for their cycle (40). Interestingly, it has been demonstrated that human immunodeficiency virus (HIV) can regulate activity of TFEB and, in consequence, autophagy in order to promote its own replication and cell survival. To this end, HIV has established a productive infection and the use of negative regulatory factor (NEF) to inhibit autophagy by binding with beclin-1 (BECN1), promoting TFEB phosphorylation via mTOR activation and cytosolic sequestration, resulting in inactivation of TFEB transcription activity and promotion of viral replication (41).

Recent observations extend the importance of TFEB to coronavirus infection. Viral proteins that induce lysosomal stress, such as SARS-CoV ORF3a or SARS-CoV-2 ORF8b, trigger TFEB nuclear translocation, linking viral pathogenesis with host transcriptional reprogramming (16, 23). This may reflect a dual role: TFEB activation can support antiviral immunity by enhancing autophagic clearance and cytokine signaling, but excessive or dysregulated activation may fuel inflammation. Thus, TFEB functions at a critical intersection of antiviral defense, cellular stress adaptation, and immune regulation, making it a promising target for therapeutic modulation in COVID-19.

## TFEB-ALP axis in COVID-19 immunopathology and Long COVID

Reinforcing TFEB’s role beyond cell−autonomous defense, recent studies place TFEB-ALP at the nexus of endosomal-lysosomal trafficking and host restriction [7,8]. In parallel, clinical and experimental data increasingly connect disruption of the ALP with the immunopathology of COVID-19 (42). This view is consistent with recent reviews that specifically discuss dysregulated autophagy in acute COVID-19 and Long COVID (43, 44), highlighting persistent alterations in TFEB-lysosome signaling as a potential driver of chronic immune activation. In severe cases, impaired autophagic flux coincides with uncontrolled inflammation, complement activation, and tissue injury. The clinical worsening of individuals with COVID-19 may be related to immunopathological damage, reflected by increased concentrations of proinflammatory cytokines such as tumor necrosis factor alpha (TNF-α) and interleukin 6 (IL-6) (45). Post-mortem and transplant analyses of lungs from patients with nonresolving COVID-19 revealed simultaneous abnormalities in autophagy markers and complement activity, supporting the concept of ALP-complement crosstalk in disease progression (46). A serious and devastating deterioration depends on the cytokine release syndrome (CRS), or “cytokine storm”, which is a consequence of the overproduction of cytokines and leads to rapid multi-organ system failure and damage of tissues in patients suffering from virus infection (47–49).

Circulating biomarkers further support this connection. In hospitalized patients, dynamic alterations of sequestosome 1 (p62/SQSTM1) and microtubule-associated proteins 1A/1B light chain 3B/autophagy-related 5 (LC3/ATG5) have been observed alongside cytokine signatures, consistent with systemic perturbation of autophagy in severe COVID-19 (50). Persistent complement activation, including elevated membrane attack complex (MAC) and iC3b fragments, has been documented for months after acute infection, suggesting that unresolved immune activation contributes to post-acute sequelae. Such chronic dysregulation is aligned with the clinical picture of Long COVID, which encompasses fatigue, cognitive dysfunction, and multi-organ involvement (51).

Recent studies further implicate mitochondrial dysfunction and metabolic exhaustion in immune cells as part of the TFEB-ALP axis dysregulation in Long COVID (52–56). Persistent TFEB activation in T lymphocytes and monocytes may reflect a compensatory attempt to restore lysosomal clearance, but paradoxically contributes to chronic inflammation and impaired cellular energetics. Autophagy-related impairment in mitophagy was associated with reduced adenosine triphosphate (ATP) availability and sustained reactive oxygen species (ROS) production, fueling neuroinflammation and fatigue. Clinical data from multiple cohorts confirm impaired mitochondrial respiration and elevated oxidative stress in Long COVID patients (53–55), while mechanistic studies show diminished mitophagy and altered expression of E3 ubiquitin-protein ligase Parkin/PTEN-induced kinase 1 related pathways (56, 57). These findings align with reports linking disrupted lysosomal-mitochondrial crosstalk to long-term sequelae of viral infections. Incorporating TFEB-ALP biomarkers into clinical monitoring may therefore provide diagnostic and therapeutic value in managing Long COVID.

The TFEB-ALP axis provides a mechanistic link between viral replication and host inflammatory responses. By restoring lysosomal biogenesis and autophagic flux, TFEB activity may counteract viral strategies that block degradation pathways. At the same time, TFEB-mediated regulation of cytokine production, inflammasome clearance, and antigen presentation positions this transcription factor as a gatekeeper of immune homeostasis. Dysregulated TFEB signaling, however, could exacerbate hyperinflammatory states and contribute to persistent pathology.

Integrating these findings, a model emerges in which TFEB-governed autophagy intersects with complement activity and systemic inflammation – strengthening the rationale for host-directed interventions that normalize autolysosomal flux rather than relying on lysosomotropic agents (13). In acute disease, this imbalance promotes tissue damage and cytokine storm; in Long COVID, it sustains low-grade inflammation and immune dysregulation. This framework highlights the importance of targeting host pathways, such as TFEB-ALP, not only for immediate antiviral defense but also for mitigating long-term sequelae of coronavirus infection.

## Therapeutic implications

Since 2019, multiple vaccines and evidence−based antivirals/biologics for COVID−19 have been authorized; nevertheless, host−directed mechanisms remain relevant. Lastly, targeting the ALP has attracted considerable interest as a host-directed strategy against COVID-19 (58). Early in the pandemic, lysosomotropic agents such as chloroquine and hydroxychloroquine were investigated for their ability to alkalinize endosomes and block viral entry (59, 60). Although these drugs proved ineffective and are no longer recommended for clinical use, they remain valuable as mechanistic probes highlighting the importance of lysosomal pH and trafficking in SARS-CoV-2 infection (61–64).

A more promising direction is the modulation of TFEB activity (10, 25, 26). Pharmacological interventions that restore TFEB nuclear translocation and autophagic flux could counteract viral blockade of APG-L fusion. By enhancing lysosomal clearance, such strategies may reduce viral persistence, normalize antigen presentation, and dampen excessive inflammatory responses. In addition, TFEB’s role in cytokine regulation and inflammasome clearance suggests that its activation might mitigate hyperinflammation and cytokine storm, both of which are drivers of severe COVID-19.

The therapeutic relevance of TFEB-ALP modulation extends beyond acute infection. Persistent complement activation and autophagy dysregulation are increasingly recognized in Long COVID, where chronic inflammation underlies multi-system symptoms. Restoring balance within the TFEB-ALP axis could therefore serve not only as an antiviral strategy but also as an approach to alleviate long-term sequelae. Biomarkers of autophagy and complement activation may assist in identifying patients most likely to benefit from such therapies (46, 50, 51).

Future directions include the exploration of small molecules or biologics that specifically enhance TFEB activity without broad lysosomotropic effects, as well as the integration of TFEB-ALP markers into clinical studies of Long COVID. Developing host-targeted interventions that recalibrate autophagy and immune homeostasis may ultimately improve both acute outcomes and post-acute recovery in COVID-19.

## Conclusion

SARS-CoV-2 infection profoundly disrupts the ALP, impairing host defense and fueling inflammation. At the center of this process lies TFEB, a master regulator of autophagy and lysosomal biogenesis that also governs key immune processes. Viral proteins block autophagic flux and alter TFEB signaling, tipping the balance toward viral persistence and immunopathology. Evidence from clinical and experimental studies indicates that TFEB-ALP dysfunction contributes not only to severe acute disease but also to the persistence of immune dysregulation in Long COVID. Therapeutic strategies aimed at restoring TFEB activity and autophagic flux hold promise for limiting viral replication, preventing tissue damage, and alleviating long-term sequelae. By integrating mechanistic insights with clinical observations, the TFEB-ALP axis emerges as a critical target for host-directed therapy. Modulating this pathway may provide a means to rebalance immune homeostasis, offering benefits across the spectrum of COVID-19, from acute infection to chronic post-viral syndromes.
